# Mental health impacts of COVID-19 in Ireland and the need for a secondary care mental health service response

**DOI:** 10.1017/ipm.2020.64

**Published:** 2020-05-27

**Authors:** Karen O’Connor, Margo Wrigley, Rhona Jennings, Michele Hill, Amir Niazi

**Affiliations:** 1 Consultant Psychiatrist, National Clinical Lead for Early Intervention for Psychosis, Health Service Executive, Ireland; 2 RISE Early Intervention for Psychosis Service & Home-Based Treatment Team, South Lee Mental Health Services, Health Service Executive, Cork, Ireland; 3 Consultant Psychiatrist, National Clinical Lead for Attention Deficit Hyperactivity Disorders, Health Service Executive, Ireland; 4 National Clinical Lead for Specialist Perinatal Mental Health Services, Health Service Executive, Ireland; 5 HSE Mental Health Clinical Programmes, Health Service Executive, Ireland; 6 Consultant Psychiatrist, University Lead for Student Mental Health and Wellbeing, University College Cork, Ireland; 7 Consultant Psychiatrist, National Clinical Advisor and Group Lead for Mental Health, Health Service Executive, Ireland

**Keywords:** COVID-19, mental health, policy

## Abstract

The COVID-19 pandemic is a global health emergency, the scale, speed and nature of which is beyond anything most of us have experienced in our lifetimes. The mental health burden associated with this pandemic is also likely to surpass anything we have previously experienced. In this editorial, we seek to anticipate the nature of this additional mental health burden and make recommendations on how to mitigate against and prepare for this significant increase in mental health service demand.

## Introduction

**Fig. 1.**
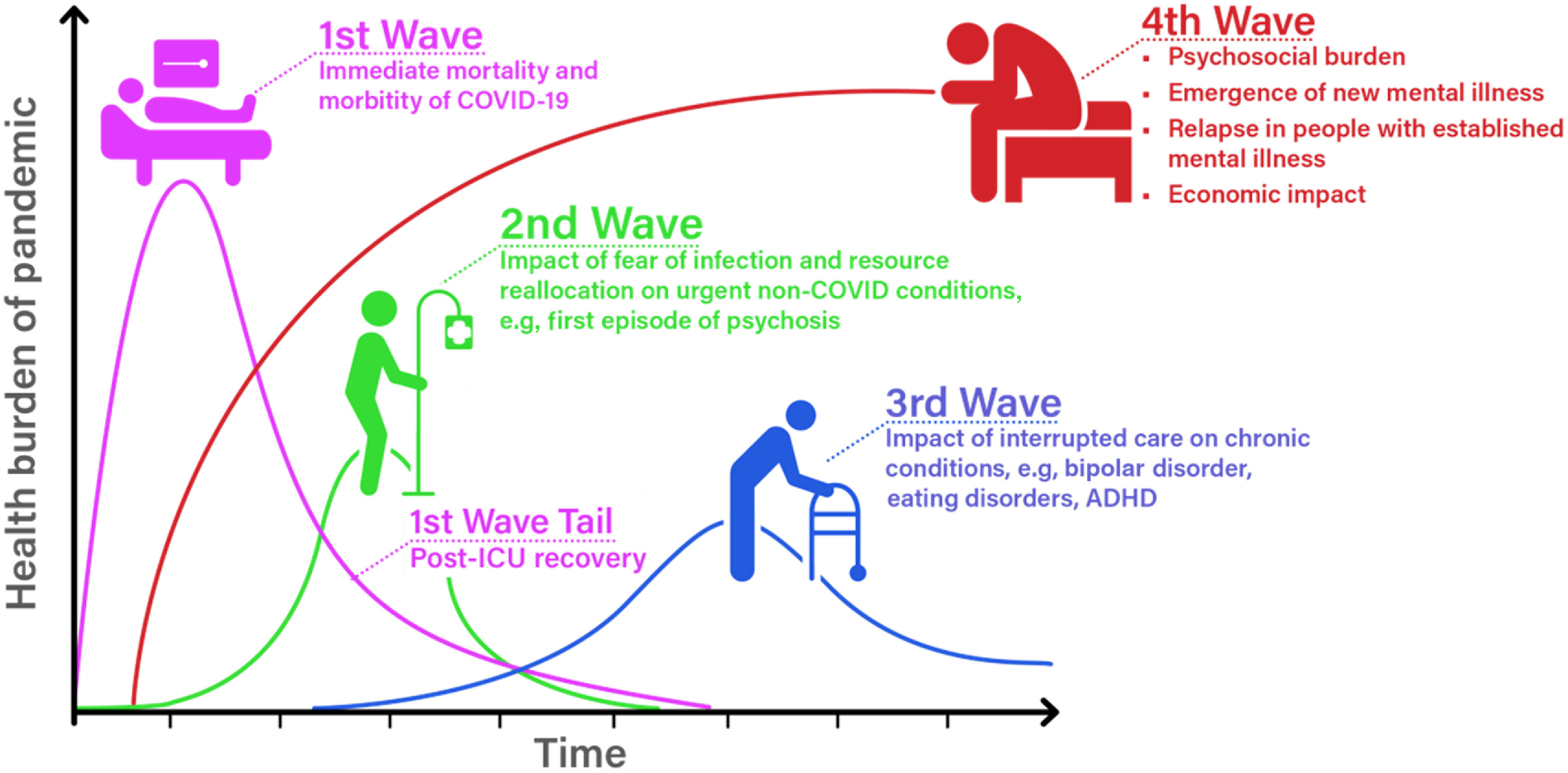
The four waves of health need associated with the COVID-19 pandemic

Source: Graph adapted from Dr Victor Tseng – Pulmonary & Critical Care Physician: University of Colorado

The psychosocial footprint associated with a major emergency is typically larger than the medical footprint. This is because the psychosocial impact extends beyond those who suffer direct medical injury to first responders, healthcare professionals delivering care to the ill, family, friends and the wider community (NATO Joint Medical Committee, 2008; Shultz *et al*. 2013; Health Service Executive, 2014). The COVID-19 pandemic is a global health emergency, the scale, speed and nature of which is beyond anything most of us, service users, healthcare staff or the general public have experienced in our lifetimes. The mental health burden associated with this pandemic is also likely to surpass anything we have previously experienced. Therefore, it is essential for Mental Health Services in Ireland to anticipate the nature of this need and plan a coordinated response to address it (see Fig. 1). To better define and plan for the mental health impact of this pandemic, an expert working group was formed in the office of the National Clinical Advisor and Group Lead for Mental Health in the Health Service Executive in Ireland. This expert working group was made of up of the authors and involved additional consultation with 14 mental health experts from across the mental health specialties including General Adult, Child and Adolescent, Intellectual Disabilities and Psychiatry of Later life.

## The four waves of health need associated with the COVID-19 pandemic

The likely timeline and nature of the various waves of health needs that will arise because of the COVID-19 pandemic are illustrated in Fig. 1. While the Irish health service must prepare in the first instance for the *first pandemic wave*, we also need to plan and mitigate against the impact of the subsequent three waves of healthcare need (see Fig. 1). The *second wave* will arise because people who are in acute need of health care, for example, myocardial infarction or first-episode psychosis forestall accessing care because of fears of COVID-19 infection or because in isolation their symptoms are not recognised. This could result in a significant increase in acute non-COVID morbidity and mortality. The *third wave* will arise from the longer-term impact on people with established health problems, for example, diabetes, eating disorder or schizophrenia not accessing routine care due to health service reconfiguration, service reduction or fears of infection. This will result in people who were stable, deteriorating over time. For example, an individual is unable to attend the diabetic clinic because it is cancelled or delayed, resulting in poorer glycaemic control. A mental health example might be where an individual with an established psychotic illness is unable to attend their weekly therapeutic group, loses their job, has their routine outpatient review rescheduled and experiences increased loneliness, isolation and a relapse of psychotic symptoms. The largest and longest *fourth wave* of healthcare need will encompass the psychosocial and mental health burden associated with this pandemic. This final tsunami will not peak until sometime afterwards (months) and will sustain for months to years after the COVID-19 pandemic itself. A proportion of this psychosocial and mental health need can be met at a community and primary care level in the first instance. However, a significant proportion will require specialist intervention from secondary care mental health services. In this editorial, we seek to describe and make recommendations on how to mitigate against and prepare for this increase in mental health service demand. However, it is important to note that any plan developed in the context of this pandemic will require review and revision as further evidence becomes available.

## Features particular to this pandemic that will result in an increased mental health burden in the medium to longer term

There are several features particular to the COVID-19 emergency that are likely to amplify and prolong both the psychosocial and the mental health burden associated with this pandemic. These features include the morbidity and mortality associated with COVID-19, the relentless media coverage, the social distancing measures, the altered pathways to access care, the changes to the care that is available, the suspension of development plans in mental health services and the economic impact on all populations in society. Table 1 describes these features in more detail.

**Table 1.** Features of this pandemic that will impact on mental health burden in the medium to longer term

Feature of the COVID-19 Pandemic	Impact on Mental Health Burden
Scale of the COVID-19 pandemic	PTSD Complex grief Depression Anxiety disorders Neuropsychiatric consequences of covid infection
Relentless media coverage	Difficult to cope with anxiety, fear and anticipation of the pandemic. Difficulty sleeping, eating, taking a break from coverage and impact.
Social distancing measures	Greater impact on vulnerable groups, for example, those in poverty, insecure housing/work, single-parent families, abusive relationships, direct provision and people with mental illness who will have less social and professional support.
Secondary economic crisis	Well-established association with higher rates of mental illness, suicide and substance use disorders
Reduced non-COVID-19 health service utilization	Reluctance to attend for acute care due to fears of COVID-19 infection resulting in delays in effective treatment and increase in crisis presentations
Reduced availability/altered access to mental health services	Reconfiguration of services and redeployment of staff results in reduced access to care
Retraction of the National Clinical Programmes	
Self-harm	Inability to meet the anticipated increase in self-harm presentations Associated with increased morbidity, mortality and increased burden on Community Mental Health Teams
Early intervention for psychosis	Failure to implement national roll out in line with Model of Care resulting in: • Increase in duration of untreated psychosis and an associated worsening of prognosis. • Failure to deliver evidence-based interventions resulting in increased relapse, increased crisis presentations, increased hospital admissions, worse health outcomes.
Eating disorders	Failure to implement national roll out in line with Model of Care resulting in: Delays in accessing service Increased reliance on costly hospital admissions and expensive out of country placements. Failure to deliver evidence-based interventions resulting in poorer prognosis, increased crisis care and increased reliance on hospital admissions
Attention deficit hyperactivity disorder in adults	Failure to implement national roll out in line with Model of Care resulting in: Little to no access to assessment and treatment in adults Misdiagnosis Increased co-morbidity: depression, anxiety, self-harm
Suspension of all development plans	No implementation of the following: Mental Health Information System Youth Mental Health Taskforce Recommendations of National Clinical Lead and Leads in each CHO 24/7 Crisis Teams Increase in staffing numbers to be in line with A Vision for Change Recommendations in 2019 Ongoing deficits in provision for persons with: Borderline personality disorder Autism spectrum disorders Substance use disorders

Note: The contents of this Table were developed by the authors as part of an expert working group in consultation with relevant stakeholders.

The social distancing measures do not impact on all equally. Those with the fewest social and economic resources to alleviate the effects of social restrictions will be impacted the most (Morgan & Rose, 2020). This includes those living in deprived areas, with insecure and or low-income jobs, insecure housing, single-parent households or abusive relationships. It also acutely affects those with existing mental health problems, whose symptoms may worsen when access to social connections and healthcare support is restricted. The economic impact of the pandemic will further exacerbate and prolong this.

The National Clinical Programmes (NCPs) in Mental Health were developed in conjunction with the College of Psychiatrists of Ireland and are nationally led programmes seeking to improve access, quality and cost of mental health care. There are also two nationally led initiatives to support the development of Perinatal Mental Health Services and Mental Health Services for people with an Intellectual Disability. These programmes were developed to address areas of known service deficit, or indeed where there was an absence of service. The continued implementation and investment in these NCPs need to be enhanced during COVID-19.

### Groups who will be particularly vulnerable to the emergence of new mental health difficulties requiring secondary care interventions

This pandemic will be associated with an increase in people presenting for the very first time with significant mental health difficulties. Several groups are likely to be particularly vulnerable.

### COVID-19 survivors

Some people who have had a severe episode of COVID-19 illness may experience high levels of psychiatric symptoms and potentially illness afterwards. At 4-year follow-up of the severe acute respiratory syndrome (SARS) pandemic of 2003 survivors in Hong Kong Ho-Bun Lam *et al*. 2009 found that 42.5% met the threshold for at least one psychiatric diagnosis as determined at a clinical interview utilising the Semi structured Clinical Interview for the Diagnostic and Statistical Manual of Mental Disorders, fourth edition (SCID-IV). The most common diagnoses were post-traumatic stress disorder (54.5%), depression (39%), somatoform pain disorder (36.4%), panic disorder (32.5%) and obsessive–compulsive disorder (15.6%).

### People bereaved during the pandemic

Family members who have lost a loved one, who were separated from loved ones who were very ill and or died may be vulnerable to developing psychiatric illness. It was estimated that 50% of family members of SARS patients experienced psychological problems (mainly depressive symptoms) and stigmatisation (Tsang *et al*. 2004).

### Frontline workers

In the SARS research, healthcare professionals were found to be particularly vulnerable to psychiatric morbidity during and after the acute pandemic wave (Wu *et al*. 2009). A study of 549 healthcare workers in Beijing, China, 3 years after the SARS epidemic found that 10% continued to experience high levels of post-traumatic stress (Wu *et al*. 2009).

### Those with fewer social and economic resources

As described previously, those living in difficult or unstable personal/housing/employment circumstances will likely experience greater mental health impact and burden. Leading theories of suicide emphasise the critical role that social connections play in suicide prevention (Reger *et al*. 2020). Individuals experiencing suicidal ideation may lack connections to other people and often disconnect from others as suicide risk rises. Social distancing itself may be a significant risk factor for an increase in self-harm and suicide for some people. The economic impact of the pandemic is becoming increasingly apparent, with unemployment rates rising dramatically, which is an established risk factor for Mental Ill Health across the lifespan. This is likely to further compound this vulnerability and increase these risks (Corcoran *et al*. 2015).

### Extremes of the population demographic

While these issues effect all age groups, there are subgroups that are likely to be more vulnerable. Older people who are at higher risk of developing a severe form of COVID-19, particularly those who have been asked to cocoon, may be experiencing more anxiety and more isolation. Disrupted routines and reduced activity levels may undermine independence, exacerbate frailty and poor health outcomes in this population. For those with dementia living at home, an incomprehensible disruption to the person’s usual routine can lead to anxiety, agitation and sleep disturbance. Not being able to leave the house may cause an extreme reaction towards well-meaning family carers causing distress to all. In nursing homes, family and friends no longer being able to visit will distress residents. This is particularly true of those who are cognitively intact who may be equally worried about their families catching COVID-19. This is especially the case if they are aware of the deaths of fellow residents from COVID-19.

Young people (aged 15–25) are already the highest risk age for developing a mental disorder, and third-level students report even higher levels of distress than their age-matched peers (Karwig *et al*. 2014; Union of Students of Ireland, 2019). A combination of accelerated brain development and the developmental task of transition to adult life and learning are some of the explanatory factors (Duffy *et al*. 2019). These pre-existing vulnerabilities are not removed by the pandemic, and fears and uncertainty about future employment and economic stability are likely to be exacerbated by the financial impact on all of society. Prior to COVID, The My World Survey 2 in 2019, a self-report survey, showed that the already high rates of depression, anxiety and self-harm in young people reported in My World Survey 1(2012) had risen even further (Dooley, 2019). Irish youths have the fourth highest suicide rate in Europe (UNICEF, 2017). COVID is likely to impact more on the mental rather than the physical health of this group. With austerity measures, separation from peers and forced quarantine with family (who in some cases may not be a safe space) are being challenges for young people. Many have had their school and college lives disrupted, their state or college exams altered or brought forward. Their already uncertain futures looking even less clear. There are of course exceptions, for example, those with social anxiety or who were experiencing bullying. However, those subgroups will likely need even further support to re-engage with society after social restrictions are lifted.

### Individuals with an intellectual disability

Individuals with intellectual disability may struggle to understand the requirements of social restrictions and may find the disruption to their routines and reduced access to usual social supports, for example, work, and day programmes as very distressing. People with autism, within the learning disability population, may be particularly impacted, as changes in routine can be incredibly challenging for them. Rates of mental ill health within the learning disability population already exceed those in the general population and the pandemic may exacerbate this further (Hughes-McCormack *et al*. 2019). Some individuals with intellectual disability live in congregated settings. Such settings may be more vulnerable to COVID-19 infection outbreak, and this may result in increased exposure to the morbidity and mortality and, therefore, opportunity to witness the impact on others of this pandemic.

### Individuals who are pregnant or in the post-partum period

The COVID-19 pandemic is associated with a combination of factors such as worry about infection, direct effects of the virus on the foetus or on an infant, visitor restrictions, social isolation, financial strain, domestic violence and grief due to loss of family members that are likely to increase the prevalence of mental health difficulties in women during the perinatal period. The impact of no visitors in the post-partum period, or of no partner being permitted during Caesarean sections during COVID-19, may be very anxiety provoking for some. Reduced social support in the post-partum period, increased economic pressure and increased risk of domestic violence are additional potential stressors in this population.

As mentioned previously, there are two nationally led clinical programmes in place to support the development of Mental Health Services for people with an Intellectual Disability and the Perinatal Mental Health Services. It is critical that in the context of COVID-19, the development of these services is fast-tracked.

## The impact of COVID-19 on people with established mental illness

People with established mental illness are likely to be particularly vulnerable to relapse, exacerbation of symptoms and impaired functioning in the context of the COVID-19 pandemic (see Table 2).

**Table 2.** A summary of the potential impact of COVID-19 on specific mental disorders requiring input from secondary mental health services

Mental Illness	Impact of COVID-19	Mental Health Consequences
Anxiety disorders	Increased anticipatory anxiety, avoidance and anxiety symptoms. Difficulty switching off, feeling overwhelmed, feeling out of control. Insomnia, altered appetite, reduced exercise, disrupted routine. Less able to engage in adaptive behaviours that reduce anxiety, for example, social connection, exercise	Increased risk of relapse of anxiety disorder symptoms including panic attacks, agoraphobia, health-related anxiety symptoms Obsessive–compulsive disorder – fear of contamination and increased compulsive behaviours, for example, handwashing, checking, routines. Increased risk of trauma relating to the experience of COVID-19 illness or witnessing impact of illness on service user, friend and family.
Affective disorders	Increased social isolation and loneliness Insomnia, altered appetite, reduced exercise, disrupted routine. Personal experience of COVID-19 in self/family or friends.	Relapse of depression Relapse of mania
Psychosis	Rates of isolation and loneliness are higher in this population at baseline. Increased difficulty accessing care due to altered pathways and increased isolation from family/friends. Those with negative symptoms will be particularly affected by the change in routines, reduced interaction and social distancing measures. Viral infection appears to be a general risk factor for psychotic disorders, and coronavirus infection may also be a specific risk factor, conferring acute and long-term risk for psychosis (Cowan, 2020). Trauma and social marginalisation are risk factors associated with longer term increased risk of psychosis (Radua *et al*. 2018).	Relapse of psychotic symptoms, for example, hallucinations, delusions. Increased duration of untreated psychosis resulting in poorer prognosis. Further impairment of social and occupational functioning, which will be difficult to re-establish after COVID-19. Difficulty/fear of accessing evidence-based interventions, for example, psychological interventions, family interventions, individual placement support, physical health interventions. Impact of telephone versus face-to-face assessments, therapeutic interventions. Potentially increased longer-term risk of psychosis in the population. Further delays in implementing the National Early Intervention for Psychosis Model of Care.
Eating Disorders	Disruption of usual routines. Fear of loss of control. Potential for increased familial stress and conflict	Relapse or exacerbation of eating disorder Difficulty accessing evidence-based interventions, for example, psychological interventions, family interventions, physical health interventions. Impact of telephone versus face-to-face assessments, therapeutic interventions Further delays in implementing the National Eating Disorders Model of Care.
Attention deficit hyperactivity disorder	Disruption to routine, reduced capacity to be active, difficulty accessing a work/school environment at home.	Reduced capacity to relax, increased restlessness and impulsivity. Increased anxiety and depression Increased self-harm Further delays in implementing the National ADHD in Adults Model of Care.
Personality disorder	Increased social isolation and loneliness Impaired sleep, altered appetite, reduced exercise	Emotional dysregulation Increased self-harm to assist managing emotions Increased substance use to assist managing emotions Increased suicide risk
Dual diagnosis	Reduced social support which is central to most addiction programmes. Reduced access to treatment groups and programmes. Societal tendency to utilise alcohol/illicit drugs to cope with stress	Risk of relapse to alcohol or substance abuse. Increased suicide risk

Note: The contents of this Table were developed by the authors as part of an expert working group in consultation with relevant stakeholders

Furthermore, people with established mental illness also have a lower life expectancy and poorer physical health outcomes compared to people in the general population (Rodgers *et al*. 2018). Risk factors associated with poorer outcomes in COVID-19 infection include smoking, diabetes, cardiovascular disease and obesity. These risk factors are all more prevalent in people with established mental illness. As such, people with established mental illness may be at risk of poorer mental health and physical outcomes in this pandemic (Cullen *et al*. 2020).

## Mental Health Service Response

Funding of mental health services in Ireland has remained consistently low, ~6% of the overall health budget (compared to 12% in New Zealand and United Kingdom) (College of Psychiatrists of Ireland, 2020). Ireland has the third lowest number of psychiatric beds in Europe (Eurostat, 2017). The staffing recommendations for mental health teams set out in A Vision for Change have never been achieved. The latest data from the Health Service Executive in December 2019 put the staffing levels of child and adolescent mental health teams, psychiatry for older persons teams and psychiatry for people with an intellectual disability, as a percentage of AVFC recommendations at 57%, 61% and 33%, respectively. Mental health services are underfunded across the board; however, there is a societal recognition of the mental health needs of young people, and yet they struggle the most to access secondary care. This is perhaps a result of the traditional adult–paediatric split, which does not match the epidemiology. It may also be a consequence of underfunded services being unable to respond to young people until conditions are much more entrenched or repeated crises have occurred.

Like the scenario faced by intensive care units at the start of this COVID pandemic, in mental health, we are starting at a low base and facing into a tsunami of mental health need. Similar to the approach taken in the acute hospitals, we need urgent investment, building of capacity and innovation to ensure that mental health services are not overwhelmed and are able to respond to service users in a timely manner.

### Ring fence an additional COVID-19 budget for Mental Health Services

Ring fence a specific budget to allow mental health services to build capacity, adapt and innovate. In line with Slaintecare, we need to have the right care, available at the right time, in the right place (Houses of the Oireachtas Committee on the future of healthcare, 2017). Redeployment of mental health staff during the acute pandemic should be minimised and only occur in very extreme and time-limited circumstances.

A ring-fenced COVID-19 research budget, within a collaborative interagency framework, should also be introduced. Services will need to adapt and transform. However, it is critical that evolving approaches are evaluated to ensure feasibility/ acceptability and that they are associated with good health outcomes for service users and their families.

Youth Mental Health services rest in the domain of primary care counselling services in Ireland, with no representation from Psychiatry, perhaps reflecting a misguided belief in the general population, and at government level, that mental illness can always be prevented. These services are ill equipped to manage the full range of presentations that seek help. Without funded vertical integration pathways and ring-fenced funding to secondary care, there is a risk that already limited funding in AMHS and CAMHS will be channelled away from where it is most needed. Even in countries with significantly more enhanced primary care Youth Mental Health services, there is a recognition that 30% of young people who present (Headspace Australia) have needs that are in excess of what can be managed there (Rickwood *et al*. 2019). The Youth Mental Health Taskforce recommended appointment of national and local YMH leads, a focus on improving mental health services in third-level institutions, and upscaling of digital interventions all of which now need to be implemented (National Youth Mental Health Taskforce, 2017).

### Digital Mental Health

The rapid upscaling of the information technology infrastructure has been a very positive consequence of COVID-19. However, access to smart phones, laptops and high-quality broadband is an issue in many areas. This needs to be addressed as a priority. We also need to adapt and develop digital health interventions, for example, psychological interventions, family interventions, peer to peer supports, physical health interventions to augment services capacity to deliver evidence based care in the context of COVID-19 (Alvarez-Jimenez *et al*. 2015). The need for electronic records and data collection systems that monitor patient outcomes should also be developed in tandem with telemedicine.

### Accelerate implementation of the NCPs in Mental Health

A specific budget to support and protect the implementation of the NCPs during COVID-19 should be identified. Adequate resourcing of these programmes will ensure that areas of the mental health service that have already been identified as severely lacking will be able to meet demand. Now is not the time to fall backwards in the delivery of high quality, accessible care. Rather we need to accelerate service transformation and to build and strengthen capacity in our mental health services.

## Conclusion

Because of COVID-19, secondary care mental health services are facing a huge escalation of mental health need. It is emerging now, will peak in a few months’ time and will last for many months to years. Now is the time to flatten this curve. Unless we anticipate, plan and invest in all our secondary care mental health services as a priority, they will be overwhelmed with terrible consequences for the mental health and economic recovery of our country.
